# Mucosal‐First Pemphigus Vulgaris With Delayed Generalized Bullous Eruption: A Case Report

**DOI:** 10.1155/crdm/3595737

**Published:** 2026-10-22

**Authors:** Ammir Abuzahra, Bahaa Mohammed Abdalwahab Khawarh, Khadija Ideas, Jihad Ibrahim Abdalwahab Alkhawarh, Diana Daghamin, Jafar M. A. Ihryzat, Ibrahim Ideas

**Affiliations:** ^1^ College of Medicine Hebron University Hebron State of Palestine hebron.edu; ^2^ Abu Al Hassan Al Kassem Hospital Yatta State of Palestine; ^3^ Faculty of Medicine Kafr El Sheikh University Kafr El Sheikh Egypt kfs.edu.eg

**Keywords:** autoimmune blistering disease, case report, diagnostic delay, oral erosions, pemphigus vulgaris, suprabasal acantholysis

## Abstract

**Background:**

Pemphigus vulgaris (PV) may initially present with persistent oral erosions and can be mistaken for infectious or inflammatory mucosal disease.

**Case Description:**

A previously healthy 43‐year‐old woman developed recurrent painful oral erosions that persisted despite multiple antifungal and antibacterial treatments. An initial biopsy from a necrotic oral ulcer was nondiagnostic. Following substantial oral improvement, she developed a generalized painful vesiculobullous eruption involving approximately 12% of the body surface area. Nikolsky sign was positive. Repeat skin biopsy demonstrated suprabasal acantholysis and blister formation, strongly supporting PV. She was treated with oral prednisolone 60 mg/day followed by a six‐week taper, together with topical clobetasol. Cutaneous lesions had clinically cleared by approximately 25 August 2026. At follow‐up on 7 September 2026, approximately 13 days after cutaneous clearance, no active oral or cutaneous lesions or documented recurrence were present.

**Conclusion:**

This case highlights the diagnostic importance of appropriate biopsy‐site selection in persistent oral erosions and the value of repeat biopsy from an active lesion. Because direct immunofluorescence and antidesmoglein serology were not performed, the diagnosis is best regarded as probable, histopathologically supported PV rather than immunopathologically confirmed PV.

## 1. Introduction

Pemphigus vulgaris (PV) is an autoimmune intraepidermal blistering disease caused predominantly by pathogenic autoantibodies against Desmoglein 3, with or without Desmoglein 1. Loss of keratinocyte adhesion produces fragile mucosal and cutaneous blisters that rupture easily and leave painful erosions [1]. Oral involvement is common and frequently precedes cutaneous disease [2]. Because intact oral bullae are rarely observed and established lesions may resemble infectious or inflammatory mucosal disorders, diagnostic delay is well recognized [3].

The diagnosis relies on clinicopathologic correlation. Routine histopathology demonstrating suprabasal acantholysis provides strong supportive evidence, whereas perilesional direct immunofluorescence (DIF) and disease‐specific serological testing, including antidesmoglein antibodies, provide important immunopathological confirmation [1, 4]. This distinction is particularly relevant when confirmatory immunopathological testing is unavailable.

Although mucosal‐first PV and diagnostic delay have been previously described [5], the distinctive features of the present case are prolonged infection‐directed treatment, an initial nondiagnostic biopsy obtained from a secondary necrotic oral lesion, apparent oral recovery followed by a generalized vesiculobullous eruption, and the diagnostic value of repeat biopsy from an active blistering lesion. The case therefore illustrates how lesion selection at biopsy can substantially influence diagnostic yield. Because DIF and antidesmoglein serology were not performed, the diagnosis is described as probable PV supported by the clinical course and routine histopathology.

## 2. Case Description

A 43‐year‐old woman with no known chronic medical illness presented with a recurrent and progressively destructive oral mucosal process. Her only reported previous surgery was cesarean delivery. She was not taking regular medications before symptom onset and had no known drug or food allergy. No relevant family history was documented.

On 15 March 2026, two small painful lesions appeared suddenly on the tongue. They were described as lentil‐sized whitish lesions with a red base and local warmth, without documented fever. She denied preceding oral trauma, tongue biting, recent dental procedures, unusual food exposure, or previous similar episodes. Within approximately two days, the process spread rapidly across the tongue and oral cavity. Pain became severe enough to limit mouth opening and prevent normal eating and drinking.

During repeated emergency and otolaryngology evaluations, the lesions were initially attributed to an infectious process. Miconazole oral gel 2% (20 mg/g), 2.5 mL applied to the oral mucosa four times daily after meals for 14 days, was used approximately from 18 to 31 March 2026. This was followed by fluconazole 200 mg orally on the first day and 100 mg orally once daily for the subsequent 13 days, approximately 1–14 April 2026. Nystatin oral suspension 100,000 IU/mL, 5 mL swish‐and‐swallow four times daily for 10 days, was subsequently administered approximately from 20 to 29 April 2026. Amoxicillin/clavulanate 875/125 mg orally every 12 h for 7 days was given approximately from 5 to 11 May 2026. Despite these treatments, improvement was transient or incomplete, and the disease repeatedly recurred and extended to the lips. At its most severe, the lips were markedly swollen and desquamated with diffuse erosions, adherent white‐yellow slough, hemorrhagic crusting, and malodor (Figures 1A and B).

**FIGURE 1:**
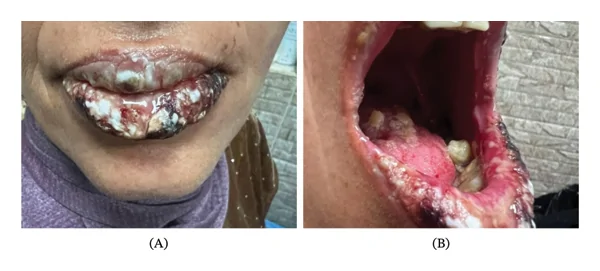
Severe oral mucosal disease during the prediagnostic phase. (A) Marked lip edema with diffuse erosions, adherent white‐yellow slough, desquamation, and hemorrhagic crusting. (B) Extensive erosive involvement of the lips and oral cavity. The appearance had repeatedly been treated as a fungal infection before autoimmune blistering disease was considered.

Laboratory testing on 2 June 2026 showed WBC 7.4 × 10^3^/µL, hemoglobin 11.8 g/dL, MCV 85.4 fL, and platelets 257 × 10^3^/µL. Vitamin B12 was 245 pg/mL, ferritin 38 ng/mL, TSH 0.91 µIU/mL, and tissue‐transglutaminase IgA 2.90 U/mL, which was negative according to the reporting laboratory. Creatinine was 1.14 mg/dL and ALT 49 U/L. ESR was 24 mm/h on 13 June.

On 18 June 2026, she was admitted because of severe oral disease and inability to tolerate oral intake. She received intravenous fluids and analgesia together with fluconazole 200 mg intravenously once daily for 5 days and amoxicillin/clavulanate 1.2 g intravenously every 8 h for 5 days. No additional systemic anti‐infective therapy was prescribed after discharge. Admission testing showed WBC 5.1 × 10^3^/µL, hemoglobin 12.7 g/dL, platelets 235 × 10^3^/µL, creatinine 0.67 mg/dL, AST 9 U/L, and alkaline phosphatase 46 U/L. High‐sensitivity C‐reactive protein was markedly elevated at 177.6 (laboratory reference 0–5), and ESR later reached 60 mm/h. Oral/throat bacterial culture demonstrated normal oral flora without pathogenic bacterial growth, while fungal culture yielded no *Candida*. No clinically relevant antimicrobial susceptibility results were reported.

An oral‐lesion biopsy obtained on 25 June and reported on 27 June 2026 contained small fragments of soft tissue showing chronic active inflammation and necrotic material without malignancy. No disease‐specific diagnosis was established. A later hospital note explicitly described the specimen as representing a secondary lesion and recommended repeat biopsy from a primary lesion.

After discharge, the oral disease improved partially but subsequently recurred. The treating team documented concern for an autoimmune process, with PV, Behçet disease, lichen planus, and other inflammatory disorders included in the differential diagnosis. Dermatology and rheumatology assessment and repeat biopsy of a primary lesion were recommended. Beginning approximately 20 July 2026, triamcinolone acetonide 0.1% dental paste was applied as a thin layer directly to the oral erosions three times daily after meals for 14 days, through approximately 2 August 2026. Lidocaine viscous 2%, 5 mL swish‐and‐spit up to three times daily as needed, was used for pain. This regimen was followed by marked improvement and progressive oral re‐epithelialization.

Approximately two days after substantial recovery of the oral lesions, the patient developed a new eruption of painful fluid‐filled vesicles and bullae that spread over the chest, abdomen, back, upper and lower limbs, and genital region. Approximately 12% of the body surface area was involved. The face, palms, soles, oral cavity, eyes, nasal mucosa, and pharyngeal mucosa were spared. Nikolsky sign was positive over actively blistering skin. No PDAI or ABSIS severity score was recorded. The eruption was not accompanied by additional systemic symptoms. Representative intact and ruptured lesions are shown in Figures 2A,B and 3.

**FIGURE 2:**
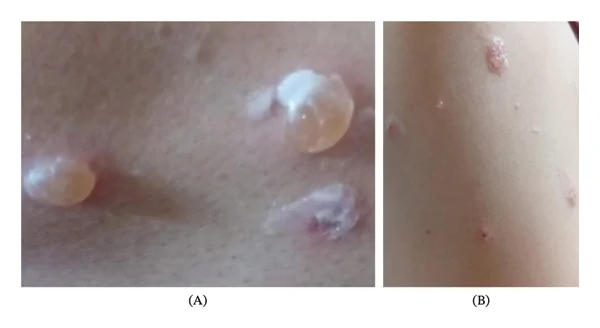
Representative lesions from the later generalized vesiculobullous eruption. (A) Multiple clear fluid‐filled vesicles/bullae, including an intact blister and a ruptured lesion. (B) Additional disseminated lesions at different stages, including postrupture erosive/healing sites. Clinically, lesions involved the trunk, limbs, and genital region while sparing the face and oral cavity.

**FIGURE 3:**
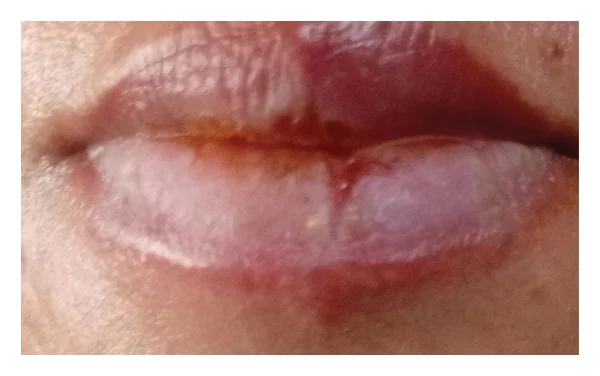
Final oral follow‐up in early September 2026 showing resolution of the previous lip and oral erosions, with no active slough, hemorrhagic crusting, or clinically significant ulceration.

Regarding potential medication‐related disease, no new systemic medication had been initiated during the 7 days immediately preceding the generalized eruption. Previous systemic antifungal and antibacterial courses had been completed several weeks earlier, while triamcinolone dental paste and topical oral analgesic treatment had been used during late July and early August. Neither dermatology nor pathology considered drug‐induced pemphigus, Stevens–Johnson syndrome/toxic epidermal necrolysis, or another bullous drug reaction to be the leading diagnosis, and no medication was discontinued because of suspected drug causality.

A skin punch biopsy was obtained around 8 August 2026 from the left lower leg. Lesional and perilesional specimens were received on 9 August and reported on 11 August. Routine histopathology showed focal spongiosis and basal vacuolar change; eosinophilic epidermal infiltration; acantholysis with wide areas of suprabasal separation and blister formation; and scattered lymphocytes, eosinophils, and acantholytic keratinocytes within the blister cavity. The dermis contained superficial and perivascular lymphocytic/eosinophilic inflammation. No malignancy was identified. The pathologist concluded that the findings were mostly in keeping with PV and recommended immunofluorescence. Representative histopathological photomicrographs were not available to the authors for inclusion in the revised manuscript. The histopathological findings reported in this case are therefore based directly on the formal biopsy reports issued by the reporting pathology laboratory.

DIF was not subsequently performed. Antidesmoglein 1 and Antidesmoglein 3 IgG testing and other autoimmune blistering serologies were also not obtained. Although DIF was recommended, the clinical and routine histopathological findings were considered sufficiently supportive at the time, and the patient demonstrated rapid clinical improvement after treatment. Consequently, the diagnosis was regarded as probable, histopathologically supported PV rather than immunopathologically confirmed PV.

Oral prednisolone was initiated on 8 August 2026 at 60 mg once daily, equivalent to three 20‐mg tablets each morning. The regimen consisted of 60 mg/day for 7 days, followed by 50 mg/day for 7 days, 40 mg/day for 7 days, 30 mg/day for 7 days, 20 mg/day for 7 days, and 10 mg/day for the final 7 days before discontinuation at approximately 18 September 2026. The total treatment duration was six weeks. Clobetasol propionate 0.05% cream was applied as a thin layer to active cutaneous lesions twice daily for 14 days. Additional treatment consisted of chlorhexidine digluconate 0.12% mouthwash, 15 mL for 30 s twice daily for 14 days; nystatin oral suspension 100,000 IU/mL, 5 mL four times daily for 14 days; calcium carbonate providing 1000 mg elemental calcium daily; and vitamin D3 800 IU once daily throughout systemic corticosteroid treatment.

The bullae progressively dried, flattened, and re‐epithelialized, with complete clinical cutaneous clearance by approximately 25 August 2026. No scarring, residual erosions, or clinically significant pigmentation was present at subsequent follow‐up. No clinically significant corticosteroid‐related adverse effects were documented, including hyperglycemia, hypertension, significant weight gain, Cushingoid features, secondary infection, gastrointestinal symptoms, or significant mood or sleep disturbance.

At the latest clinical follow‐up on 7 September 2026, approximately 13 days after cutaneous clearance, the patient had no active oral or cutaneous lesions and no documented recurrence. She was receiving prednisolone 20 mg/day as part of the planned taper at that visit. No oral recurrence or new cutaneous bullae had occurred after treatment initiation. Continued dermatology follow‐up was planned, with rituximab to be considered if relapse, corticosteroid dependence, or inadequate disease control developed. Azathioprine or mycophenolate was not initiated because clinical disease control had been achieved.

### 2.1. Diagnostic Assessment

The overall clinicopathological picture strongly favored PV. The disease began with persistent painful oral erosions, remained refractory to multiple appropriately administered antifungal and antibacterial treatments, subsequently evolved into a generalized vesiculobullous eruption, and demonstrated suprabasal acantholysis with blister formation on repeat skin biopsy [1, 6]. During the generalized eruption, approximately 12% of the body surface area was affected, Nikolsky sign was positive, and lesions involved the chest, abdomen, back, extremities, and genital region while sparing the face, palms, soles, and oral, ocular, nasal, and pharyngeal mucosa. No standardized PDAI or ABSIS disease‐severity score had been recorded.

An infectious explanation became increasingly unlikely. During the June hospitalization, oral/throat bacterial culture showed normal oral flora without pathogenic bacterial growth, and fungal culture showed no *Candida*. Moreover, the mucosal disease had failed to respond sustainably to repeated antifungal and antibacterial treatment.

Bullous pemphigoid was considered in the differential diagnosis of the generalized eruption, but the suprabasal level of cleavage with acantholysis favored PV over the subepidermal separation characteristic of bullous pemphigoid [1, 6]. Mucous membrane pemphigoid was less favored because of the subsequent widespread cutaneous eruption, lack of ocular involvement or mucosal scarring, and the histological cleavage pattern. Behçet disease was less supported by the absence of recurrent genital aphthae, ocular inflammation, and other characteristic systemic manifestations. Erosive lichen planus was less favored because typical reticular or striated lesions were absent.

Drug‐induced pemphigus and other bullous drug eruptions were also considered because the patient had received several medications during the preceding months. However, no new systemic medication had been initiated within the 7 days immediately preceding the generalized eruption, and the systemic antifungal and antibacterial courses had been completed several weeks earlier. Neither dermatology nor pathology identified a specific medication as a suspected trigger, and no medication was withdrawn because of suspected drug causality. These findings made an acute medication‐associated bullous eruption less favored, although it cannot be excluded with absolute certainty in the absence of immunopathological confirmation. The principal differential diagnoses and their relationship to the findings in this patient are summarized in Tables 1 and 2.

**TABLE 1:** Chronological summary of clinical findings, investigations, treatment, and response.

Date/period	Clinical findings	Investigations	Treatment	Response
15–17 Mar 2026	Two painful whitish tongue lesions rapidly progressed to diffuse oral involvement with severe pain and impaired oral intake	Initial clinical assessment	Supportive management	Rapid progression
18–31 Mar 2026	Persistent oral erosions	Fungal infection initially suspected	Miconazole oral gel 2%, 2.5 mL four times daily for 14 days	No sustained response
1–14 Apr 2026	Persistent/recurrent oral disease	Clinical reassessment	Fluconazole 200 mg orally on Day 1, then 100 mg/day for 13 days	Transient/incomplete improvement
20–29 Apr 2026	Recurrent oral erosions	—	Nystatin 100,000 IU/mL, 5 mL four times daily for 10 days	No sustained response
5–11 May 2026	Persistent oral/lip disease	Infectious cause remained suspected	Amoxicillin/clavulanate 875/125 mg orally every 12 h for 7 days	No sustained response
2–13 Jun 2026	Severe persistent oral disease	CBC largely preserved; ferritin, B12, TSH, and tTG‐IgA nondiagnostic; ESR 24 mm/h	—	Persistent disease
18–23 Jun 2026	Hospitalized because of severe oral erosions and inability to tolerate intake	hs‐CRP 177.6; ESR later 60 mm/h; bacterial culture normal oral flora; fungal culture negative for *Candida*	IV fluids/analgesia; fluconazole 200 mg IV daily and amoxicillin/clavulanate 1.2 g IV every 8 h for 5 days	Partial improvement
25–27 Jun 2026	Persistent oral disease	Oral biopsy: chronic active inflammation and necrosis; no malignancy; nondiagnostic	—	Repeat biopsy from a primary lesion subsequently recommended
20 Jul–2 Aug 2026	Relapsing oral/lip erosions; autoimmune blistering disorder increasingly suspected	Dermatology/rheumatology assessment	Triamcinolone acetonide 0.1% dental paste three times daily for 14 days; lidocaine viscous 2% as needed	Marked oral improvement and re‐epithelialization
∼7‐8 Aug 2026	Generalized painful vesicles/bullae involving chest, abdomen, back, limbs, and genital region; approximately 12% BSA; positive Nikolsky sign; face and oral cavity spared	Clinical assessment	Prednisolone initiated 8 Aug at 60 mg/day; clobetasol 0.05% twice daily	Progressive improvement
8–11 Aug 2026	Active generalized eruption	Left lower‐leg biopsy: suprabasal separation, acantholysis, blister formation, and acantholytic keratinocytes; DIF recommended but not performed	Prednisolone taper; clobetasol, nystatin, chlorhexidine, calcium/vitamin D	Histopathology strongly supportive of PV
By ∼25 Aug 2026	No active cutaneous bullae	Clinical follow‐up	Prednisolone taper continued	Clinical cutaneous clearance
7 Sep 2026	No active oral or cutaneous disease	Clinical follow‐up	Prednisolone 20 mg/day at this stage of taper	No documented recurrence; no significant steroid‐related adverse effects
∼18 Sep 2026	Six‐week corticosteroid course completed	—	Final prednisolone dose 10 mg/day for 7 days, then discontinued	Continued dermatology follow‐up planned

**TABLE 2:** Major differential diagnoses considered in the generalized vesiculobullous eruption.

Differential diagnosis	Typical distinguishing features	Findings in the present case
Pemphigus vulgaris	Fragile mucocutaneous bullae/erosions; suprabasal acantholysis; positive intercellular DIF	Favored by prolonged mucosal‐first disease, positive Nikolsky sign, generalized blistering, and suprabasal acantholysis; DIF/serology unavailable
Bullous pemphigoid	Usually tense bullae with subepidermal separation	Less favored because histopathology showed suprabasal rather than subepidermal separation
Mucous membrane pemphigoid	Predominantly mucosal disease; subepithelial separation; possible ocular or mucosal scarring	Less favored by generalized cutaneous disease, lack of ocular/scarring involvement, and suprabasal acantholysis
Behçet disease	Recurrent oral and genital aphthae with possible ocular and systemic manifestations	Less supported because recurrent genital aphthae, ocular disease, and characteristic systemic features were absent
Drug‐induced pemphigus/bullous drug eruption	Temporal relationship to a potentially causative medication; clinical and histologic pattern varies by disorder	Considered because of prior medication exposure, but no new systemic medication was started within 7 days before the eruption, previous systemic anti‐infective courses had ended weeks earlier, and no specific drug trigger was identified clinically

Routine histopathology strongly supported PV but did not provide definitive immunopathological confirmation. DIF of perilesional tissue, Antidesmoglein 1 and Antidesmoglein 3 serology, and other autoimmune blistering serologies were not performed. Accordingly, the final diagnosis is described as probable, histopathologically supported PV rather than immunofluorescence‐confirmed PV.

### 2.2. Therapeutic Intervention and Follow‐Up

Before recognition of an autoimmune blistering disorder, the patient received sequential courses of miconazole, fluconazole, nystatin, and amoxicillin/clavulanate, including intravenous fluconazole and amoxicillin/clavulanate during the June hospitalization, without sustained clinical response. Greater oral improvement followed topical triamcinolone acetonide 0.1% dental paste administered three times daily for 14 days.

Following development of the generalized eruption, oral prednisolone was initiated on 8 August 2026 at 60 mg/day. The dose was tapered weekly from 60 to 50, 40, 30, 20, and finally 10 mg/day, for a total treatment duration of six weeks, with discontinuation around 18 September 2026. Clobetasol propionate 0.05% cream was applied twice daily to active lesions for 14 days. Nystatin oral suspension and chlorhexidine mouthwash were administered for 14 days, and calcium plus vitamin D supplementation was continued throughout systemic corticosteroid therapy.

Cutaneous lesions had clinically cleared by approximately 25 August 2026. At the latest clinical follow‐up on 7 September 2026, approximately 13 days after cutaneous clearance, the patient was receiving prednisolone 20 mg/day and had no active oral or cutaneous lesions, residual erosions, scarring, or clinically significant pigmentation. No recurrence had been documented, and no clinically significant corticosteroid‐related adverse effects were observed. Continued dermatology follow‐up was planned. Rituximab was reserved for possible relapse, corticosteroid dependence, or inadequate disease control, while no additional steroid‐sparing agent was initiated during the documented remission.

## 3. Discussion

This case demonstrates a clinically important progression from prolonged mucosal disease to generalized cutaneous blistering. Although oral involvement commonly precedes cutaneous PV, the diagnosis in this patient was complicated by repeated treatment for presumed infection and by an initial biopsy obtained from a secondary necrotic oral lesion [2, 3, 7]. The subsequent development of widespread vesicles and bullae prompted repeat biopsy and substantially increased diagnostic confidence.

The contrast between the two biopsies represents the central diagnostic lesson of this case. Routine histopathological examination should preferably use tissue obtained from a recent intact vesicle or bulla, or from the advancing margin of an active lesion, where suprabasal acantholysis is more likely to remain preserved [8]. Accordingly, the initial biopsy in this case was probably nondiagnostic because it sampled a secondary necrotic ulcer, whereas the subsequent biopsy from an active blister clearly demonstrated suprabasal separation and acantholysis. In patients with suspected autoimmune blistering disease, a two‐biopsy approach is appropriate: lesional tissue is obtained for routine histopathological examination, while a separate perilesional specimen is submitted for DIF [1, 4]. In the present case, DIF was recommended after the second biopsy but was not subsequently performed.

The generalized eruption also introduced diagnostic uncertainty because the patient had been exposed to several medications during the preceding months. Drug‐induced pemphigus, other bullous drug eruptions, bullous pemphigoid, and mucous membrane pemphigoid therefore warrant consideration. However, no new systemic medication had been initiated during the 7 days immediately preceding the eruption, and the previous systemic antifungal and antibacterial courses had been completed several weeks earlier. Neither dermatology nor pathology identified a specific medication as a suspected trigger. In addition, the prolonged mucosal‐first course, positive Nikolsky sign, and histological demonstration of suprabasal acantholysis favored PV. Pemphigoid disorders were less compatible with the suprabasal level of cleavage, as they characteristically demonstrate subepidermal separation [1, 6]. Nevertheless, medication‐associated disease cannot be excluded with absolute certainty in the absence of complete immunopathological confirmation.

Routine histopathology in this case provides strong supportive evidence for PV but does not independently establish definitive immunopathological confirmation in every clinical context. DIF of perilesional tissue and Serum antidesmoglein 1 and 3 antibody testing were not performed. This limitation is particularly relevant because these investigations are useful both for confirming the diagnosis and for distinguishing PV from other autoimmune blistering disorders [1, 4]. Accordingly, we describe the diagnosis as probable, histopathologically supported PV rather than definitively confirmed PV.

The patient responded rapidly to systemic corticosteroid therapy. Prednisolone was initiated at 60 mg/day and tapered weekly over six weeks, together with topical clobetasol. Clinical cutaneous clearance was documented by approximately 25 August 2026, and no active oral or cutaneous lesions were present at follow‐up on 7 September, 13 days later. No clinically significant corticosteroid‐related adverse effects or relapse had been documented by that visit. However, this postclearance observation period was short and should not be interpreted as evidence of sustained long‐term remission. Continued dermatology surveillance was therefore planned, with rituximab reserved for relapse, corticosteroid dependence, or inadequate disease control [9–11].

The principal limitations of this report are the absence of DIF and antidesmoglein serology and the relatively short duration of follow‐up following cutaneous clearance. Representative histopathological photomicrographs were also not available for inclusion. The original pathology material remained under the custody of the reporting pathology laboratory and was not available to the authors for preparation of publication‐quality images during revision; therefore, the histopathological descriptions presented here are based directly on the formal biopsy reports. Because DIF was not performed, no DIF images were available. These limitations reduce diagnostic certainty despite the strong clinical and routine histopathological evidence supporting PV.

## 4. Conclusion

Persistent oral erosions that fail conventional anti‐infective treatment should prompt early consideration of autoimmune blistering disorders. This case highlights the importance of selecting an appropriate active lesion for routine histopathology and obtaining separate perilesional tissue for DIF. Although repeat biopsy demonstrated suprabasal acantholysis strongly supporting PV, the absence of DIF and antidesmoglein serology limits the diagnosis to probable, histopathologically supported PV. The patient achieved short‐term clinical clearance without documented recurrence at the latest follow‐up; however, longer surveillance is necessary to determine whether sustained remission is maintained.

### 4.1. Learning Points

• Oral PV may precede cutaneous blistering by months and can be mistaken for recurrent candidiasis.
• Failure of repeated anti‐infective therapy should trigger diagnostic reassessment rather than continued empiric treatment.
• A necrotic ulcer bed may yield nonspecific pathology; an active lesion plus perilesional tissue is far more informative.
• Suprabasal acantholysis with blister formation strongly supports PV and helps distinguish it from pemphigoid disorders.
• DIF and antidesmoglein serology remain important for formal confirmation, even when clinicopathologic evidence is compelling.

## Author Contributions

Ammir Abuzahra: conceptualization, data curation, investigation, project administration, visualization, writing–original draft, and writing–review and editing. Bahaa Mohammed Abdalwahab Khawarh, Khadija Ideas, Jihad Ibrahim Abdalwahab Alkhawarh, Diana Daghamin, and Jafar M. A. Ihryzat: investigation, data curation, and writing–review and editing. Ibrahim Ideas: project administration, and writing–review and editing.

## Funding

The authors received no specific funding for this work.

## Disclosure

All authors approved the final manuscript and agree to be accountable for the work.

## Ethics Statement

Ethical approval was obtained from Abu Al Hassan Al Kassem Hospital Institutional Review Board, Approval ID: HKY126. This case report was conducted in accordance with the ethical principles of the Declaration of Helsinki. The case was prepared and reported in accordance with the CARE (CAse REport) guidelines [12].

## Consent

Written informed consent was obtained from the patient for publication of this case report and the accompanying images.

## Conflicts of Interest

The authors declare no conflicts of interest.

## Patient Perspective

The patient described the oral phase as the most disabling part of the illness because pain prevented normal eating and drinking and repeatedly returned after treatment. The sudden later appearance of blisters across the body was distressing, but she reported progressive drying and disappearance of the lesions after the dermatology‐directed regimen. At the latest follow‐up, she reported no active oral or cutaneous lesions and no recurrence since treatment initiation.

## Data Availability

The de‐identified clinical data supporting this case are contained within the article. Additional de‐identified source information may be available from the corresponding author on reasonable request, subject to patient privacy and institutional requirements.
